# Comparison of the Four Proposed Apgar Scoring Systems in the Assessment of Birth Asphyxia and Adverse Early Neurologic Outcomes

**DOI:** 10.1371/journal.pone.0122116

**Published:** 2015-03-26

**Authors:** Hosein Dalili, Firouzeh Nili, Mahdi Sheikh, Amir Kamal Hardani, Mamak Shariat, Fatemeh Nayeri

**Affiliations:** 1 Maternal, Fetal and Neonatal Research Center, Vali-asr Hospital, Tehran University of Medical Sciences, Tehran, Iran; 2 Breastfeeding Research Center, Vali-asr Hospital, Tehran University of Medical Sciences, Tehran, Iran; Vanderbilt University, UNITED STATES

## Abstract

**Objectives:**

To compare the Conventional, Specified, Expanded and Combined Apgar scoring systems in predicting birth asphyxia and the adverse early neurologic outcomes.

**Methods:**

This prospective cohort study was conducted on 464 admitted neonates. In the delivery room, after delivery the umbilical cord was double clamped and a blood samples was obtained from the umbilical artery for blood gas analysis, meanwhile on the 1- , 5- and 10- minutes Conventional, Specified, Expanded, and Combined Apgar scores were recorded. Then the neonates were followed and intracranial ultrasound imaging was performed, and the following information were recorded: the occurrence of birth asphyxia, hypoxic Ischemic Encephalopathy (HIE), intraventricular hemorrhage (IVH), and neonatal seizure.

**Results:**

The Combined-Apgar score had the highest sensitivity (97%) and specificity (99%) in predicting birth asphyxia, followed by the Specified-Apgar score that was also highly sensitive (95%) and specific (97%). The Expanded-Apgar score was highly specific (95%) but not sensitive (67%) and the Conventional-Apgar score had the lowest sensitivity (81%) and low specificity (81%) in predicting birth asphyxia. When adjusted for gestational age, only the low 5-minute Combined-Apgar score was independently associated with the occurrence of HIE (B = 1.61, P = 0.02) and IVH (B = 2.8, P = 0.01).

**Conclusions:**

The newly proposed Combined-Apgar score is highly sensitive and specific in predicting birth asphyxia and also is a good predictor of the occurrence of HIE and IVH in asphyxiated neonates.

## Introduction

Asphyxia is a damaging condition of impaired blood gas exchange, leading, if it persists to progressive hypoxemia, hypercapnia, and tissue oxygen debt, which can cause serious central nervous system, respiratory, cardiovascular, and renal complications.[1]

The prevalence of fetal asphyxia is increasing; in 1990s the incidence of asphyxia was reported as 5.4 per 1000 live births,[2] while in the 2006 the American College of Obstetricians and Gynecologists (ACOG) reported an incidence of 25–73 per 1000 live births.[1]

Early newborn assessment, prediction of the neonatal complications, and prompt intervention is crucial, to prevent the progression of asphyxia and also not to loose the window of therapeutic opportunity for minimizing its complications.[3]

Apgar scoring system which was described in 1950s, is the oldest and most commonly used assessment tools for the evaluation of the newborn and the need for interventions in the delivery room.[4] In addition some studies used the Apgar score for the assessment of birth asphyxia and the prediction of adverse neonatal outcomes.[5–7]

The ACOG in the committee opinion on Apgar score, stated that a low Apgar score beyond 5 minutes is a suggestive criterion for asphyxia, however, there are several limitations with the Apgar score that make it inappropriate to be used alone for establishing the diagnosis of asphyxia, or for predicting the adverse neonatal outcomes.[8] Apgar score is influenced by the gestational age, neonate's maturity, drugs, etc. In addition the Apgar score that is assigned during resuscitation and intubation does not give a precise assessment of the newborn's situation.[9] These limitations made the researchers think of an alternative to the conventional Apgar score. Therefore the Specified-Apgar and later the Expanded-Apgar scores were suggested to allow the assessment of the newborn's condition independent of the interventions and the gestational age. [8,10,11] Despite these advances, there was still a need for a more comprehensive and precise scoring system that could predict the occurrence of adverse neonatal outcomes. Thus the Combined-Apgar score was proposed by Rüdiger et al. that consists of both the Specified and the Expanded Apgar scores to allow a more detailed description of neonate's postnatal condition.[12]

Despite all the attempts, there is still a considerable gap in this area of research; by our extensive search we could not find published studies evaluating the four proposed scoring systems and comparing them in the assessment of the newborns. No study has evaluated the sensitivity, specificity and the cutoffs of these scoring systems in assessing birth asphyxia and adverse neonatal outcomes especially after the corrections made to the limited conventional Apgar score. To date there is no accepted standards for evaluating the newborns (especially the preterm and the resuscitated newborns) under clinical conditions in the delivery room.[8,13]

We therefore conducted this study to compare the sensitivity and specificity of the four proposed Apgar scoring systems in the assessment of birth asphyxia and in predicting adverse early neurologic outcomes in the neonates especially after asphyxia and the hemodynamic instability of the newborns in the delivery room.

## Materials and Methods

### Study population and study design

This prospective cohort study was conducted on the newborns admitted to a tertiary referral hospital with an annual birth rate of 2200 births, between September 2012 and February 2014. Inclusion Criteria were: live birth at a gestational age > 25 weeks, birth within the study center, and the requirement for hospital admission. A total of 596 newborns were eligible to participate in the study, and 132 were excluded due to the following criteria: gestational age at ≤ 25 weeks, birth out of the study center, major congenital anomalies, death in the delivery room, and missing parental informed consent. A written informed consent was taken from the parents before the delivery. A total of 464 neonates born at the gestational ages of 26–40 weeks completed the study which was approved by the Research Deputy and the Ethics Committee of the Tehran University of Medical Sciences.

### Data and specimen collection

**Fig 1:**
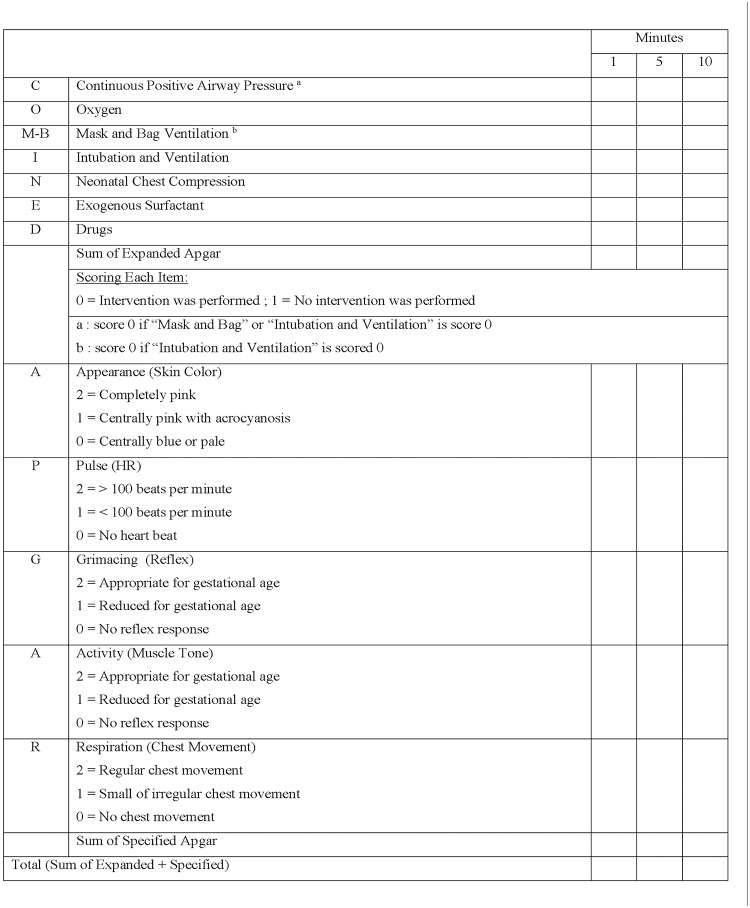
The Combined-Apgar scoring system, consists of the Expanded and Specified Apgar scoring systems. In 2012 the Combined-Apgar score was introduced by Rudiger et al.

Upon enrollment, a standardized questionnaire was completed for every neonate through medical records and physical examinations. The questionnaire contained inquiries regarding the gestational age at birth as calculated based on the ultrasound imaging, type of delivery, birth weight, and neonatal gender. In the delivery room, after the neonate was delivered, the umbilical cord was double clamped, and immediately a blood sample was obtained from the umbilical artery using a heparinized syringe for arterial blood gas analysis, which was performed by a blood gas analyzer (Nova Biomedical, Waltham, MA), meanwhile on the 1- , 5- and 10- minutes the Conventional, Specified, Expanded, and Combined Apgar scores were recorded by educated physicians according to Table 1 and Fig. 1. [4,8,12] The conventional Apgar score ranges from 0–10, the Specified Apgar score ranges from 0–10, the Expanded Apgar score ranges from 0–7, and the Combined Apgar score ranges from 0–17. The physicians were all educated in the delivery room prior to the beginning of the study to assure the consistency and avoid interpersonal biases of calculating the four proposed Apgar scores. Then the neonates were followed by a neonatologist until discharge and the following information were recorded: the occurrence of birth asphyxia (that was diagnosed based on the following criteria: 1. a sentinel (signal) hypoxic event occurring immediately before or during labor, 2. evidence of a metabolic acidosis in fetal umbilical cord arterial blood obtained at delivery (pH ≤ 7.00 and base deficit ≥ 12 mmol/L), 3. onset of multisystem involvement (central nervous, renal, pulmonary, cardiovascular, gastrointestinal systems) within 72 hours of birth, 4. exclusion of other identifiable etiologies, such as trauma, coagulation disorders, infectious conditions, or genetic disorders), [14,15] the occurrence and severity of Hypoxic Ischemic Encephalopathy (HIE) (that was diagnosed based on the following criteria: 1. a sentinel (signal) hypoxic event occurring immediately before or during labor, 2. evidence of a metabolic acidosis in fetal umbilical cord arterial blood obtained at delivery (pH ≤ 7.00 and base deficit ≥ 12 mmol/L), 3. early onset of neonatal encephalopathy as detected by structured neurologic examination performed serially by a single investigating neonatologist at approximately 12, 36, and 72 hours after birth. 4. exclusion of other identifiable etiologies, such as trauma, coagulation disorders, infectious conditions, or genetic disorders), also the severity of HIE was recorded based on Sarnat chart (Table 2) [1,14–16], and the occurrence of neonatal seizure that was documented by an abnormal electroencephalogram (EEG) performed in case of clinical seizures and in every comatose infant at 16–48 hours after birth, which was not due to electrolyte imbalances or any identifiable infectious, hypoglycemic, or traumatic causes.[15] In addition intracranial ultrasound imaging was performed on all the neonates by a single person during the first postnatal week to detect the occurrence of intraventricular hemorrhage (IVH) and its severity.

**Table 1:** The Conventional-Apgar scoring system as introduced by Virginia Apgar in 1953.

Sign	The Score		
	0	1	2
**Heart rate**	Absent	Less than 100	More than 100
**Respiration**	Absent	Slow, irregular	Good, Crying
**Muscle tone**	Limp	Some flexion	Active motion
**Reflex Irritability**	No response	Grimace	Cough, sneeze, cry
**Color**	Blue or pale	Pink body, blue extremities	Completely pink

**Table 2:** The Sarnat chart for the staging of the severity of hypoxic ischemic encephalopathy as introduced by Sarnat H.B and Sarnat M.S. in 1976.

Severity	Stage 1 (Mild)	Stage 2 (Moderate)	Stage 3 (Severe)
**Level of consciousness**	Hyperalert	Lethargic or Obtunded	Stupor or coma
**Activity**	Normal	Decreased	Absent
**Neuromuscular Control**			
Muscle Tone	Normal	Mild hypotonia	Flaccid
Posture	Mild distal flexion	Strong distal flexion	Intermittent decerebration
Stretch Reflexes	Overactive	Overactive	Decreased or absent
**Complex or primitive reflexes**			
Suck	Weak	Weak or absent	Absent
Moro (Startle)	Strong	Weak	Absent
Tonic neck	Slight	Strong	Absent
**Autonomic Function**			
Pupils	Mydriasis	Miosis	Variable
Heart Rate	Tachycardia	Bradycardia	Variable
**Seizures**	None	Common	Uncommon

### Statistical analysis

All statistical analyses were performed using SPSS statistical software (version 18.0.0: PASW, Chicago, IL). Chi-squared analysis, Fisher's exact test, independent-samples T test, One-way Anova, Pearson correlation coefficient, and ROC curve were used to analyze the correlations and relationships between variables. Multivariate logistic regression was used to evaluate the dependency of the obtained results. Sample size was calculated for a power of 80% and an alpha error of 0.05. Estimated odds ratios (ORs) with 95% confidence intervals (95% CIs) and P value < 0.05 were used to evaluate the statistical significance of the associations and correlations between variables.

## Results

### Descriptive statistics

This prospective cohort study was conducted on 464 admitted neonates who were born at gestational ages > 25 weeks. At enrollment the mean ± standard deviation (SD) for gestational age at birth was 35.9 ± 3.2 weeks; birth weight was 2586 ± 866 grams; cord blood pH was 7.25 ± 0.09; cord blood bicarbonate was 19.2 ± 4 mEq/l; cord blood base deficit was 2.2 ± 4 mEq/l; Conventional-Apgar score at 5- minute was 8.8 ± 1.1 (Range 3–10); Specified-Apagr score at 5- minute was 8.6 ± 1.6 (Range 3–10); Expanded-Apgar score at 5-minute was 5.7 ± 1.6 (Range 0–7); and for Combined-Apgar score at 5- minute was 15 ± 3.1 (Range 4–17).

Of 464 neonates who completed the study, 246 were preterm (born at a gestational age < 37 weeks) (53%), 209 had low birth weight (birth weight < 2500 grams) (45%), 43 had birth asphyxia (9.2%), 29 had hypoxic ischemic encephalopathy (6.25%) that was mild in 6 neonates (0.9%), moderate in 14 (3%) and severe in 9 neonates (1.9%). IVH was detected in 41 of the neonates (8.8%), and seizure with an abnormal EEG occurred in 35 neonates (7.5%).

### Birth asphyxia, umbilical cord blood gas and the proposed scores

There was a significant linear correlation between all the proposed Apgar scores and the cord blood pH, and base deficit, and an inverse correlation with PCO2 levels. However the Combined-Apgar score had the strongest correlations with cord blood gas parameters.(Table 3)

**Table 3:** Pearson correlation coefficient showing the association between different Apgar scores and cord blood gas parameters.

The Scoring System	pH	PO2	PCO2	HCO3	Base Deficit
**Conventional Apgar**					
	r = 0.13	r = 0.07	r = - 0.11	r = 0.14	r = - 0.23
	P = 0.008 *	P = 0.14	P = 0.03 *	P = 0.7	P = 0.000 *
**Expanded Apgar**					
	r = 0.18	r = 0.13	r = - 0.11	r = - 0.05	r = - 0.29
	P = 0.000 *	P = 0.01 *	P = 0.03 *	P = 0.27	P = 0.000 *
**Specified Apgar**					
	r = 0.24	r = 0.11	r = - 0.16	r = - 0.04	r = 0.31
	P = 0.000 *	P = 0.02 *	P = 0.002 *	P = 0.37	P = 0.000 *
**Combined Apgar**					
	r = 0.31	r = 0.12	r = - 0.2	r = 0.02	r = 0.32
	P = 0.000 *	P = 0.01 *	P = 0.000 *	P = 0.61	P = 0.000 *

*: P value less than 0.05

**Fig 2:**
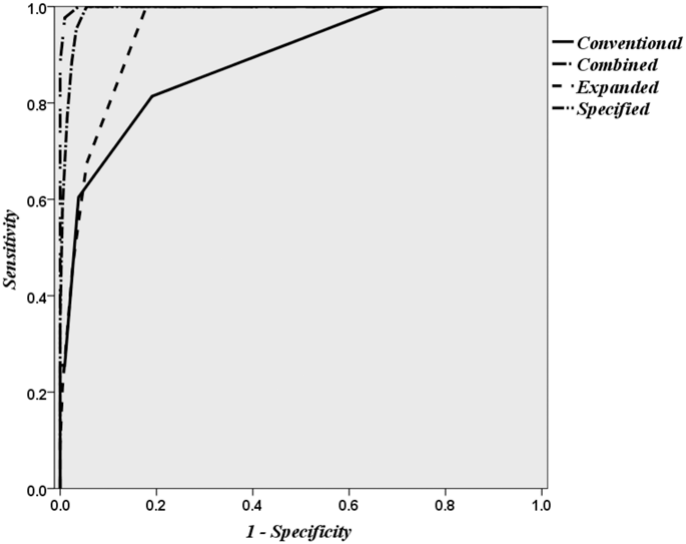
ROC curve showing the sensitivity and Specificity of Conventional, Specified, Expanded and Combined Apgar scores in predicting birth asphyxia.

Fig. 2 illustrates the sensitivity and specificity of the 5-minute proposed scores in predicting birth asphyxia by using a receiver operating characteristic (ROC) curve. Area under the curve for Conventional-Apgar was 0.88, for Expanded-Apgar was 0.94, for Specified-Apgar was 0.99 and for Combined-Apgar was 0.99.(Fig. 2)(Table 4)

**Table 4:** Comparison of the four proposed Apgar scores in predicting birth asphyxia.

	Sensitivity	Specificity	PPV	NPV	LR +	LR -	AUC	95%CI
**Conventional Apgar score (cutoff less than 7)**	81%	81%	73.3%	92.8%	4.26	0.23	0.887	0.83–0.94
**Expanded Apgar score (cutoff less than 4)**	67%	95%	55.7%	96.5%	13.4	0.34	0.949	0.92–0.97
**Specified Apgar score (cutoff less than 7)**	95%	97%	79.1%	98.7%	31.6	0.05	0.991	0.98–0.99
**Combined Apgar score (cutoff less than 10)**	97%	99%	93.1%	99.7%	97	0.03	0.999	0.99–1.00

PPV: Positive Predictive Value

NPV: Negative Predictive Value

LR +: Likelihood Ration for positive results

LR-: Likelihood Ration for negative results

AUC: Area Under the Curve

95%CI: 95% Confidence Interval

Also we calculated the best cutoff for each 5- minute calculated scores in predicting birth asphyxia with the observed sensitivity, specificity, positive predictive value (PPV), and negative predictive value (NPV). (Table 4)

As illustrated in Fig. 2, and Table 4. the Combined-Apgar score has the highest sensitivity, specificity, PPV and NPV in predicting birth asphyxia.

15 neonates (3.2%) had a 5- minute Conventional-Apgar score < 7, 52 neonates (11.2%) had a 5-minute Expanded-Apgar score < 4, 46 neonates (9.9%) had a 5- minute Specified-Apgar score < 7, and 48 neonates (10.3) had a 5- minute Combined-Apgar score < 10.

### The proposed scores in predicting adverse early neurologic outcome

In the asphyxiated neonates, among the proposed scores, the low 5- minute Combined-Apgar score (less than 10) (P = 0.01) and low 5- minute Expanded-Apgar score (less than 4) (P = 0.02) were the only scores associated with the occurrence of HIE, however neither could predict the severity of HIE. The low 5- minute Combined- Apgar score (P = 0.001) and low 5- minute Expanded-Apgar score (less than 4) (P = 0.01) were the only scores associated with the occurrence of IVH, however neither could predict the severity of IVH. None of the low 5- minute scores was associated with the occurrence of neonatal seizure. When adjusted for gestational age by using logistic regression model, only low 5-minute Combined-Apgar score remained significantly associated with HIE (B = 1.61, P = 0.02) and IVH (B = 2.8, P = 0.01).

## Discussion

In the current study the newly proposed Combined-Apgar score was highly sensitive and specific in predicting birth asphyxia, with a high PPV and NPV. In addition a low 5- minute Combined-Apgar score was significantly associated with major adverse early neurologic outcomes in the asphyxiated neonates, which was independent of gestational age.

This study is among the very first studies that compare the four proposed scoring systems in assessing the newborns in the delivery room. In this study the Combined-Apgar score had the highest sensitivity and specificity in predicting birth asphyxia, followed by the Specified-Apgar score that was also highly sensitive and specific. The Expanded-Apgar score was highly specific but not sensitive and the Conventional-Apgar score had the lowest sensitivity and low specificity in predicting birth asphyxia. The low sensitivity and specificity of the Conventional-Apgar scoring system in assessing birth asphyxia was recognized in many studies since 1980s; Skyes et al. in a study of 1210 newborn stated that because the Apgar score doesn’t usually reflect the degree of acidosis at delivery, its value in asphyxial assessment must be questioned.[17] In addition due to the serious limitations of the Conventional-Apgar score especially in premature and resuscitated neonates, many papers criticized the use of the Apgar score as the gold standard in assessing asphyxia and neonatal outcomes.[18,8,9,13,14] Even a Lancet paper had called for the Apgar score to be pensioned off.[18] Neil Marlow in his paper indicated that in the absence of prematurity and neonatal resuscitation a low Apgar score may identify adverse neonatal outcome, therefore suggested using Apgar score with caution while continue to search for an alternative for the Apgar score that could be used in all the newborns.[19] To solve the limitations of the Coventional-Apgar score, two additional scoring systems were suggested; Rudiger et al. suggested the Specified-Apgar score, that contained the same but slightly changed items of the Conventional-Apgar score, it describes the condition of the newborn regardless of the medical interventions or the gestational age.[10] Further, for more precise neonatal evaluation the ACOG and AAP introduced the Expanded-Apgar score that counts the medical intervention given to the newborn in the delivery room.[8] In 2012 Rudiger et al. introduced the Combined-Apgar scoring system that is a combination of both Specified and Expanded Apgar scores, and documented that the Combined-Apgar score was superior to Specified and Expanded Apgar scores alone in predicting neonatal mortality in preterm infants.[12]

Based on the results of the current study for the first time cutoff points were suggested for the 5- minute Combined, Expanded and Specified Apgar scoring systems for the assessment of birth asphyxia and adverse neonatal outcome. A 5- minute Conventional-Apgar score < 10, Expanded-Apgar score < 4 and Specified-Apgar score < 7 were associated with birth asphyxia and severe neonatal acidosis.

In our study only a low 5-minute Combined-Apgar score could independently predict the occurrence of HIE and IVH in asphyxiated neonates, however it could not predict the severity of HIE or HIV. None of the Conventional, Specified, or Expanded Apgar scores alone could independently predict the adverse neurologic outcomes in asphyxiated neonates. This was in accordance with the ACOG and AAP committee opinion on the Conventional-Apgar score and also other studies that indicated a Conventional-Apgar score at 5- minute correlates poorly with future neurologic outcomes and therefore should not be used as a predictor of neurologic or adverse neonatal outcomes.[7,8,14,18–21]

## Conclusions

This study shows that the newly proposed Combined-Apgar score that was introduced in 2012 by changing and combining the Conventional, Specified and Expanded Apgar scores, to solve the limitations of using each of these scores alone, is highly sensitive and specific in predicting birth asphyxia with a high PPV and NPV, and also is a good predictor of the occurrence of HIE and IVH in asphyxiated neonates.
